# Clone Mapper: An Online Suite of Tools for RNAi Experiments in *Caenorhabditis elegans*

**DOI:** 10.1534/g3.114.013052

**Published:** 2014-09-02

**Authors:** Nishant Thakur, Nathalie Pujol, Laurent Tichit, Jonathan J. Ewbank

**Affiliations:** * Centre d’Immunologie de Marseille-Luminy, UM2 Aix-Marseille Université, Case 906, 13288 Marseille Cedex 9, France; † INSERM U1104, 13288 Marseille, France; ‡ CNRS UMR7280, 13288 Marseille, France; § Institut de Mathématiques de Marseille, Site Sud, Campus de Luminy, Case 907 13288 Marseille Cedex 9, France

**Keywords:** database, algorithm, gene discovery, functional genomics, MPscan

## Abstract

RNA interference (RNAi), mediated by the introduction of a specific double-stranded RNA, is a powerful method to investigate gene function. It is widely used in the *Caenorhabditis elegans* research community. An expanding number of laboratories conduct genome-wide RNAi screens, using standard libraries of bacterial clones each designed to produce a specific double-stranded RNA. Proper interpretation of results from RNAi experiments requires a series of analytical steps, from the verification of the identity of bacterial clones, to the identification of the clones’ potential targets. Despite the popularity of the technique, no user-friendly set of tools allowing these steps to be carried out accurately, automatically, and at a large scale, is currently available. We report here the design and production of Clone Mapper, an online suite of tools specifically adapted to the analysis pipeline typical for RNAi experiments with *C. elegans*. We show that Clone Mapper overcomes the limitations of existing techniques and provide examples illustrating its potential for the identification of biologically relevant genes. The Clone Mapper tools are freely available via http://www.ciml.univ-mrs.fr/EWBANK_jonathan/software.html.

RNA interference (RNAi) is a powerful and widely used method to investigate gene function. Researchers using the model nematode *Caenorhabditis elegans* often use a feeding method for RNAi that involves culturing worms on a bacterial clone expressing a double-stranded RNA (dsRNA) that is intended to target a specific worm gene (Timmons *et al.* 2001; Timmons and Fire 1998). Because worms can be handled robotically, screens can be automated and large numbers of clones tested in parallel (Squiban *et al.* 2012). Collections of RNAi clones are available. One made by the Ahringer lab contains polymerase chain reaction (PCR)-amplified fragments of genomic DNA (Kamath *et al.* 2003), whereas the library made by the Vidal lab (Rual *et al.* 2004) was constructed from ORFeome clones, which are derived from cDNA (Reboul *et al.* 2001). Part of the strength of the method arises from the fact that knowledge of the sequence of the dsRNA in principle allows the corresponding target gene(s) to be identified.

In common with any large-scale resource, the available bacterial RNAi clone libraries contain errors (*e.g.*, clone positions inverted on 96-well plates). For the Ahringer library, this error rate is estimated to be approximately 7% (http://www2.gurdon.cam.ac.uk/~ahringerlab/pages/rnai.html; Qu *et al.* 2011). These can be compounded by handling errors during a screen, resulting in error rates as high as 15% (Pukkila-Worley *et al.* 2014). This means that clones need to be checked by sequencing to confirm their identity. Interpreting the sequences, to confirm clone identity, can be laborious when dealing with large numbers of clones.

In *C. elegans* long dsRNAs (often >1 kb) are used, in contrast to the short interfering RNAs (siRNA; typically 19−25 bp long) used in vertebrates. Each dsRNA can thus give rise to a multitude of siRNAs, which complicates target identification. Many published studies have relied on the assignment of targets provided by the community database Wormbase (Yook *et al.* 2012). This currently suffers from a number of limitations (Wormbase release WS242). The first is that target identification is based on empirical criteria. The sequence of a “primary target” is at least 95% identical with the clone insert sequence for at least 100 nucleotides (Fievet *et al.* 2013); for “secondary targets” the definition is more than 80% identity for greater than 200 nucleotides (Kamath and Ahringer 2003). These figures are calculated using BLAT (Kent 2002), which is not perfectly adapted to the task for algorithmic reasons (Imelfort 2009). Further, the target(s) of a given clone are predicted assuming that all RNAi clones contain an insert derived from genomic DNA (Figure 1A). This assumption is clearly incorrect when applied to Vidal clones generated from intron-containing genes and can lead to overprediction of clone targets (Figure 1B). At the same time, no secondary targets are predicted for Vidal RNAi clones within Wormbase currently, leading to underprediction of clone targets.

**Figure 1:**
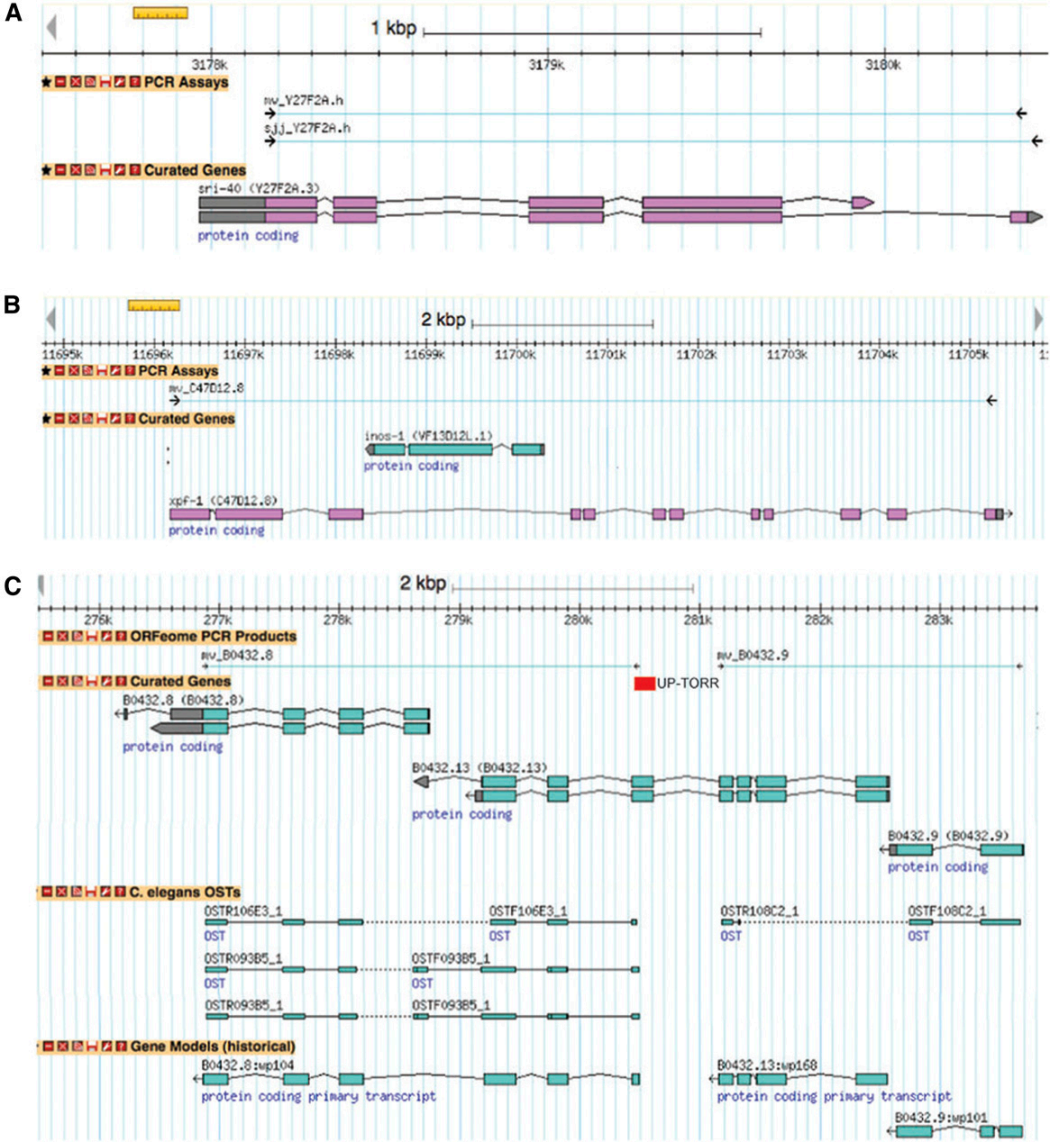
Limitations of current RNAi clone annotation illustrated with edited screen grabs from the Wormbase genome browser (WS242). (A) Wormbase currently reports RNAi clone sequences on the basis of genomic DNA, so that sjj_Y27F2A.h and mv_Y27F2A.h are associated with essentially identical insert sequences. (B) Wormbase consequently erroneously reports intronic genes as cDNA clone targets. In the case shown here, contrary to current Wormbase annotation, *inos-1* cannot be a target of mv_C47D12.8. (C) For certain cDNA-derived clones, the genomic positions of oligonucleotide primer pairs, designed on the basis of a historical gene model, do not correspond to a current gene model. For the left-hand ORFeome polymerase chain reaction (PCR) product, mv_B0432.8, the gene model used when the primer pair was designed is shown (B0432.8:wp168), but for the adjacent mv_B0432.9, the model is unavailable. In some cases, as shown here for the clones mv_B0432.8 and mv_B0432.9, the current gene models may require revision since there is conflicting ORF-sequence tag (OST) evidence. The extent of the PCR product predicted by UP-TORR on the basis of mv_B0432.8 primer sequences is indicated by the red rectangle. The reason for this erroneous prediction is not clear.

A tool, UP-TORR, has been developed that partially resolves these issues (Hu *et al.* 2013). As discussed herein, it too has some drawbacks. UP-TORR is designed for researchers using RNAi in different model systems (human, mouse, *Drosophila*, *C. elegans*) and so lacks some basic species-specific functions. For example, the standard *C. elegans* RNAi clone names (with prefixes “sjj_” and “sjj2_” or “mv_” for the Ahringer or Vidal library clones, respectively) cannot be used as input to UP-TORR. It is also not well adapted to the analysis of large datasets derived from genome-wide screens. We therefore decided to construct a tool specifically for *C. elegans*, basing target identification on matching fragments of sequence generated *in silico* from the predicted inserts of RNAi clones. This is part of a collection of tools, called Clone Mapper, that also allow clone verification and sequence retrieval. It is publically available via http://www.ciml.univ-mrs.fr/EWBANK_jonathan/software.html.

## Materials and Methods

### Data sources

The reference genome sequence and transcript sequences (WS235 and WS240) were downloaded from ftp://ftp.wormbase.org/pub/wormbase/species/c_elegans/sequence/. Following the Wormbase convention, transcripts corresponding to coding genes were used for the target library; those corresponding to coding genes and pseudogenes were used for the clone insert library. RNAi reagent information was extracted from the GFF3 file at ftp://ftp.wormbase.org/pub/wormbase/species/c_elegans/gff/. Since the original ORFeome primer sequences were designed (Reboul *et al.* 2001), there have been changes in the reference sequence of the *C. elegans* genome, most recently for release WS235 (see http://www.wormbase.org/about/wormbase_release_WS235). For some 500 ORFeome products, the original primer sequences no longer match to the genome (K. Howe, personal communication). New (pseudo)-primer sequences designed for these products (incorporating the change present in the WS235 genome sequence) were kindly provided by K. Howe; the relevant file is available on request.

To extract the clone-target gene pairs established by Wormbase (WS235), primary targets were retrieved from ftp://ftp.wormbase.org/pub/wormbase/species/c_elegans/annotation/pcr_product2gene; a list of secondary targets was kindly provided by C. Grove.

### Clone-target identification

To identify potential targets of RNAi clones, we first generated all possible 21 bp fragments from the predicted sequence of each RNAi clone insert and then we searched for matches between these fragments and transcript sequences (see Figure 4B). To rank RNAi clone-target transcript pairs, we calculated a score for each pair using a simple formula:

$$\mathrm{Score}/100=\left(\mathrm{MOS}/10\right)\left(\mathrm{MOS}/\mathrm{POS}\right){\left(\mathrm{MNO}/\mathrm{PNO}\right)}^{2}$$

The different parameters are defined as follows:

PNO: Possible nonoverlapping segments; maximum number of nonoverlapping segments of length *l* that can be generated from the clone insert. By default, *l* = 21 bp.
POS: Possible overlapping segments; maximum number of overlapping segments of length *l* that can be generated from the clone insert.
MNO: Matched nonoverlapping segments; number of nonoverlapping segments that are found in the targets transcript sequence; with a perfect match MNO = PNO; with no match MNO = 0.
MOS: Matched overlapping segments; number of overlapping segments that are found in the targets transcript sequence; with a perfect match MOS = POS; with no match MOS = 0.

In the score, weight is given to the MOS on the assumption that the absolute number of fragments generated from the RNAi clone insert that perfectly match a target transcript influences the probability that the target transcript will be affected. This value is divided by 10 to compensate for the inappropriate weight that would otherwise be assigned to perfect matches of small transcripts to large RNAi clone inserts. The MOS/POS ratio represents the overall sequence similarity between an RNAi clone insert and its target transcript. The more similar they are, the greater the ratio. The MNO/PNO element derives from the assumption that if different siRNAs produced by a clone insert match sequences within the target transcript, then there will be a greater chance of the target transcript being knocked down compared with when siRNAs produced from a single region of a clone insert match only one or a few sequences within the target transcript. The adjusted weight given to the MNO/PNO ratio reflects the assumption that RNAi will be more efficient when siRNAs are generated from multiple nonoverlapping segments that have the potential to target different nonoverlapping regions of a transcript.

The score was given a constant threshold of 100, so that if the calculated score exceeded 100, it was adjusted to 100. The equation for the score can be rearranged to:

$$\mathrm{Score}=10{\left(\mathrm{MNO}\times \mathrm{MOS}\right)}^{2}/{\left(\mathrm{POS}\times \mathrm{PNO}\right)}^{2}\leq 100.$$

### Software

For clone mapping, the BLAST program from the National Center for Biotechnology Information (Altschul *et al.* 1990) was locally installed and run with default parameters. Target mapping used MPScan (Rivals *et al.* 2009), with default parameters. For the comparison with published datasets of RNAi screens, when necessary, lists of target genes were updated to WS240 using Wormbase Converter (Engelmann *et al.* 2011). Network analysis used the GeneMania plugin (version 2012-08-02-core; Montojo *et al.* 2010; Saito *et al.* 2012) within Cytoscape (v2.8.1) (Shannon *et al.* 2003; Smoot *et al.* 2011). Programs for the various tools of Clone Mapper were written in Perl and the user interface was developed using HTML, PHP, JavaScript, and MySQL.

## Results

### Construction of an *in silico* library of RNAi clones

**Figure 2:**
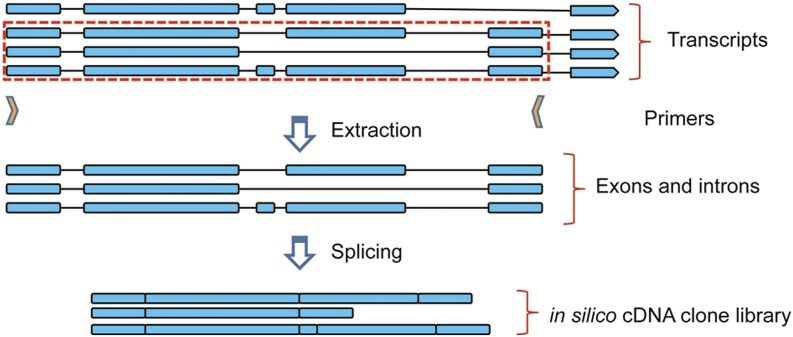
Schematic representation of the *in silico* construction of a library of cDNA-derived RNAi clone inserts. The genomic coordinates of each primer were compared to those of exons in a library of predicted transcripts. For each transcript that could potentially be amplified by a given pair of primers the corresponding sequence was extracted and spliced *in silico*.

Wormbase is the repository for a wealth of genetic, genomic, and bibliographic information. There are, however, some lacunae, such as the fact that the DNA inserts of cDNA-derived RNAi clones are not available. We therefore first constructed libraries of sequences corresponding to the expected inserts of the clones contained within the Ahringer genomic (Kamath *et al.* 2003) and Vidal cDNA-derived (Rual *et al.* 2004) RNAi collections. For the former, we also included a supplementary set of 3507 clones that recently became available. With the exception of this set, the primers made to amplify clone inserts were designed more than a decade ago. Since then, there have been minor changes in the genome sequence and more extensive changes in gene structure prediction. To correct the former problem, Wormbase calculates pseudo-primer sequences to ensure a perfect alignment between primer and genome sequence (C. Grove, personal communication). Since the Ahringer clones contain genomic inserts, generating insert sequences was relatively straightforward. The relevant coordinates were extracted from the publicly available General Feature Format (gff) file on the Wormbase ftp site and used to retrieve the corresponding genomic sequence for all of the clones. The Vidal RNAi clones are generated from the ORFeome collection. Having extracted the coordinates of the distal end of each mapped oligonucleotide primer pair (kindly provided by K. Howe, Wormbase), we calculated the proximal coordinates using the known length of each primer. The genomic coordinates of each primer were then compared with those of each transcript in an *in silico* transcript library to identify all transcripts that could potentially be amplified by a given pair of primers (Figure 2; see the section *Materials and Methods*).

For close to 15% of the clones in the Vidal library, primer pairs do not match current gene models. In the example shown in Figure 1C, the primer pair mv_B0432.8 was designed on the basis of a single gene model that existed until 2003. The predicted exons of this gene were subsequently assigned to 2 genes (B0432.8 and B0432.13). The insert sequence of clones like mv_B0432.8 that do not correspond to current gene models cannot thus be readily predicted *in silico* and we excluded these clones. This resulted in a library of 18,405 transcripts from 13,792 genes, corresponding to 88.2% of all ORFeome clones and 85.9% of the Vidal collection of 11599 RNAi clones. These sequences are available via Clone Mapper (see the section *A tool for clone verification*).

### A tool for clone verification

**Figure 3:**
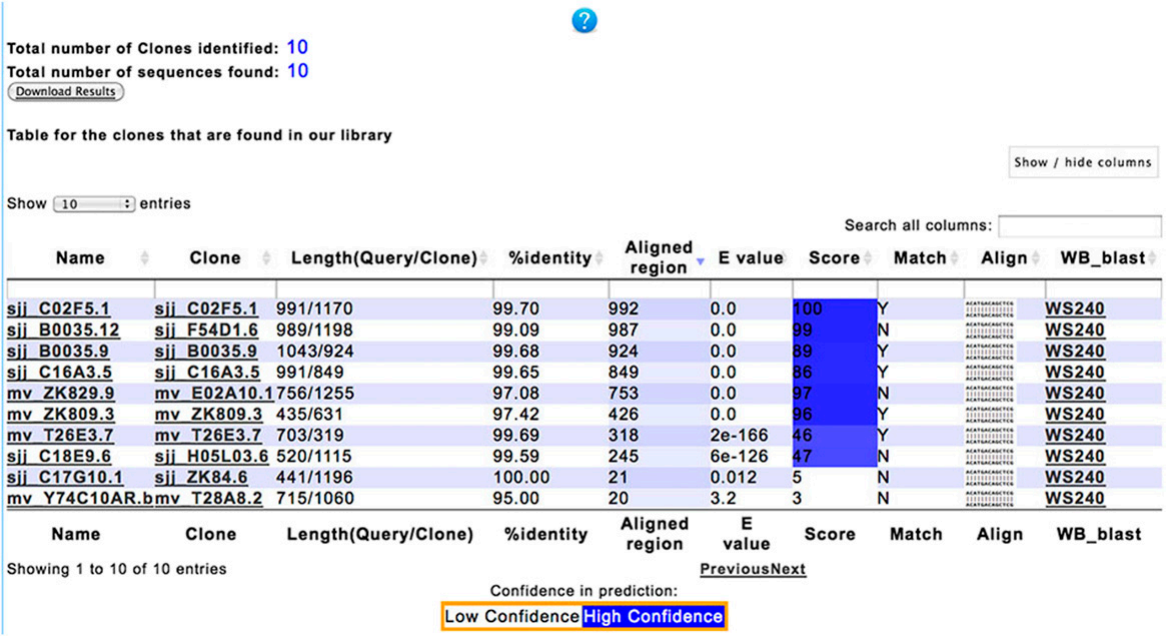
An example of RNAi clone identification using Clone Mapper. The DNA sequences obtained upon sequencing of 10 RNAi clones, from (Zugasti *et al.* 2014), were used as input into Clone Mapper. The results obtained, ranked by “Aligned region,” are shown in this screen-grab. The leftmost column shows the library name of each clone, the next column the name of the clone that best matches the experimentally determined RNAi clone insert sequence. In this example, half the clones appeared to be what was expected; for 3 of 5 of the others, an alternative identity was assigned with high confidence. For the remaining clones only a very short sequence matches a clone in the *in silico* library. These sequences can be compared directly to the genome of *C. elegans* by clicking the link in the rightmost column. The exact meaning of the different columns and options is explained in the help document, accessible by clicking the question mark at the top of the screen.

Given the errors that are intrinsic to any large collection of clones, it is indispensable to verify that RNAi clones selected through screens correspond to what they are supposed to be. This is generally done by resequencing and comparing the obtained sequence to the genome of *C. elegans* and crosschecking the position with that expected for the clone. Checking in this way becomes laborious when one needs to sequence-verify tens or hundreds of clones. We therefore made a BLASTN-based tool to match experimentally determined clone sequences with our *in silico* clone sequence libraries. It returns an output showing whether the clone is the expected one, and if not what the clone is most likely to be (Figure 3). This became the first tool in a suite that we have called Clone Mapper and for which we provide a web-based access via www.ciml.univ-mrs.fr/EWBANK_jonathan/software.html. The other functionalities are described below.

### Identifying potential targets of RNAi clones

**Figure 4:**
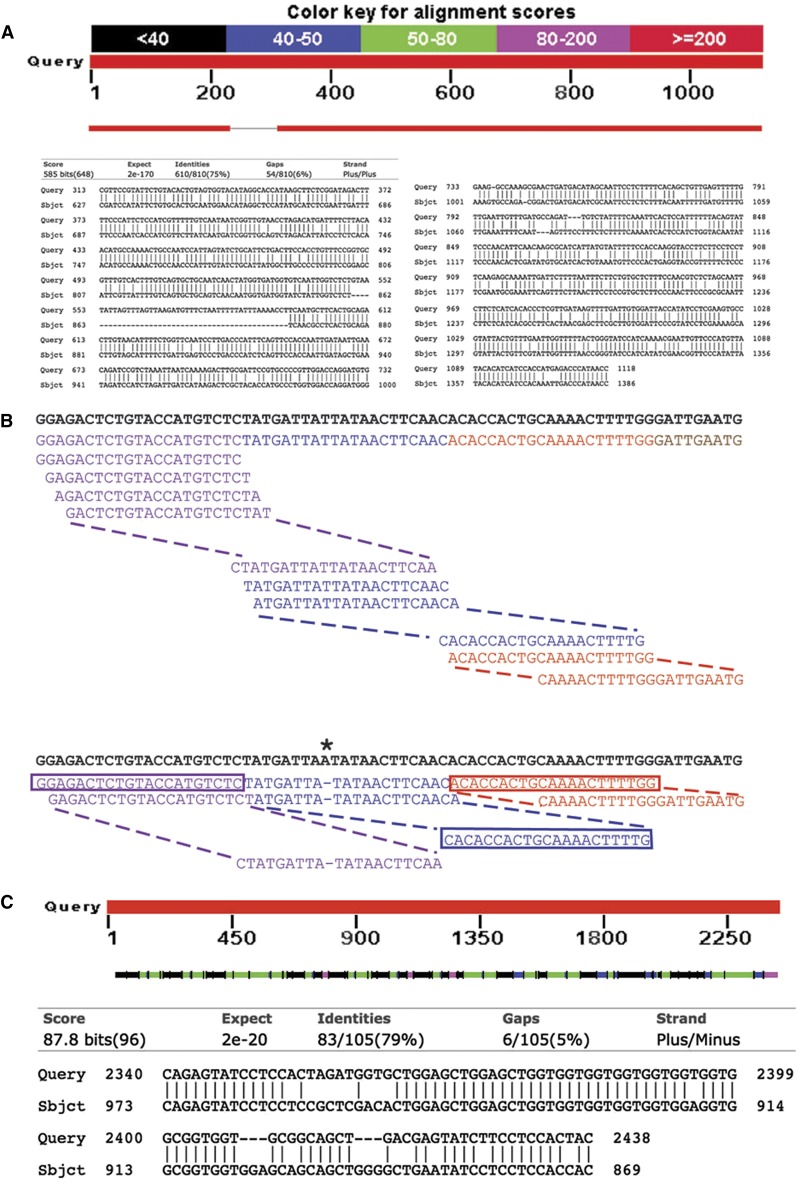
Basis of the target identification strategy. (A) An example of a target identified by Wormbase but not Clone Mapper. The RNAi clone-transcript pair (sjj_B0222.9 - F15E6.6) displays overall high identity, with blocks of >200 nucleotides with >80% identity, but does not contain a single 21 bp contiguous stretch of identical sequence. (B) The approach implemented in Clone Mapper for defining targets of RNAi clones. The set of possible nonoverlapping 21mer fragments (PNO) are generated starting from the 5′ end of each predicted clone insert sequence (in black). In the example shown, there are 3 complete (purple, blue, red) and one partial (brown) PNOs. All possible overlapping 21mer segments (POS) are generated and assigned to the corresponding PNO; for simplicity only a selection of POS are shown for each PNO. The library of all transcripts is queried with each POS to identify matched overlapping segments (MOS). In the example shown in the lower part of the panel, a transcript (in bold) with a single difference from the clone insert sequence (*) is shown. The number of PNOs that contain at least one POS that exactly matches a given transcript is counted (MNO; here 3). An example of one matching POS for each PNO is boxed. A score is then calculated (see *Materials and Methods*). (C) An example of a target identified by Clone Mapper but not Wormbase. Here the RNAi clone insert sequence has multiple stretch of sequence that have perfect matches over more than 21 nt to a target transcript (upper part of the panel; sjj_Y50E8.g and ZK643.8a), but no contiguous region of 100 nt with 95% identity (lower part of the panel; the longest stretch of identity in the selected alignment of one fragment shown here is 31 nt).

Given the shortcomings of current target prediction (see above; Figure 4A), and given the known molecular basis of RNAi, we next sought to design an alternative approach based on matching short clone-derived sequences against a comprehensive collection of predicted transcript sequences. In *C. elegans*, dsRNA gives rise to siRNAs of different sizes (19−28 bp); 22 bp is the predominant length, approximately 20% are 21mers, and <10% are shorter than 21 bp (Gent *et al.* 2010). In Clone Mapper, therefore, the clone sequence is diced *in silico* into fragments of a predetermined size. By default Clone Mapper uses 21mers, corresponding to >90% of the *in vivo* siRNAs. Increasing the oligomer size would restrict the number of potential targets identified, whereas, as discussed below, decreasing the oligomer size would allow more potential targets to be captured, but at the probable expense of increasing the proportion of false-positives. The number of occurrences of each oligomer within each transcript is then counted, and a score (from 0 to 100, with 100 corresponding to a high confidence target) assigned on the basis of a simple formula (Figure 4B; see the section *Materials and Methods*). The method allows the identification of potential targets that would not otherwise be found (*e.g.*, Figure 4C).

The predicted targets (protein coding genes) for all of the Ahringer and Vidal RNAi clones in our library have been precomputed and can be retrieved by entering a clone name in Clone Mapper. Alternatively, a user can input any sequence and its potential target transcript (protein coding and/or noncoding) will be calculated *de novo*. Conversely, the identity of clones predicted to target a given gene, or set of genes, can be retrieved by entering the relevant identifiers in the query box under the “Find targets” rubric.

### A comparative analysis of potential targets

**Figure 5:**
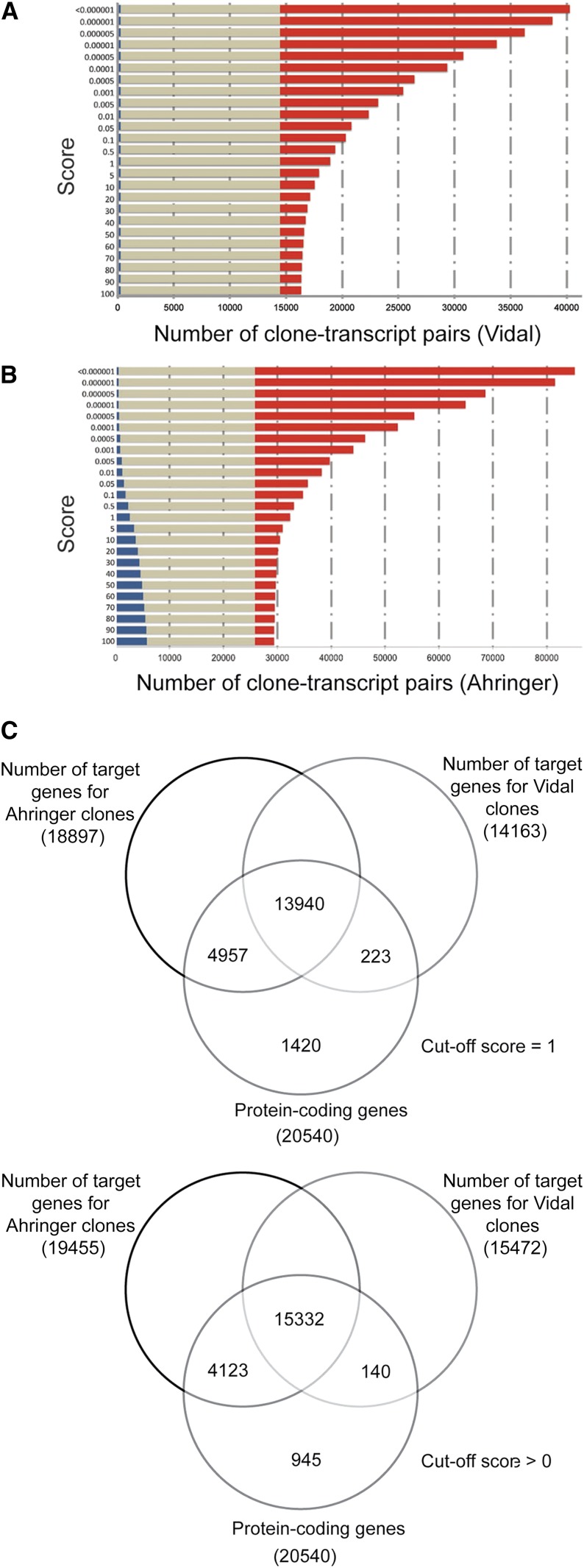
Target coverage with Clone Mapper. Comparison of the coverage of RNAi clone – target pairs for targets identified only with Clone Mapper (red), only by Wormbase (WS235; blue) or both (brown) at different cut-off scores for the Vidal (A) and Ahringer (B) clone collections. (C) Number of protein-coding genes identified by Clone Mapper as potential targets for the Vidal and Ahringer RNAi clones using 2 different scores (1 and the less stringent >0, upper and lower parts of the panel respectively) compared to the total number of predicted protein-coding genes (20540; WS240).

To establish on a genome-wide scale how different the transcript to RNAi clone correspondences obtained with Clone Mapper were from those reported in Wormbase, we conducted a global comparative analysis. We compared the overlap between Wormbase and Clone Mapper predictions across a range of scores for each transcript-RNAi clone pair. With regards the Vidal RNAi clones, even at the greatest scores, Clone Mapper predicted essentially all (98%) of the Wormbase-predicted clone-target pairs (Figure 5A). The missing fraction all falls into the category of overpredicted (Figure 1C). On the other hand, 1865 clone-target pairs not reported in Wormbase were found. Relaxing the stringency (decreasing the cut-off score from the maximum of 100) progressively increased this number; using a cut-off score of ≥1, there were 4482, which represents an increase of 30% over the total number of Wormbase predicted clone-target pairs (Figure 5A). For the Ahringer RNAi clones, when the analysis was performed with the maximum cut-off score of 100, 5825 (22.5%) of the Wormbase-predicted clone-target pairs were not found by Clone Mapper, whereas an additional 3552 were found by Clone Mapper alone. In this case, reducing the cut-off score progressively increased both the overlap between the two sets and the number of novel clone-target pairs (Figure 5B). With a cut-off score of ≥1, there were 2581 and 6539 clone-target pairs specific to Wormbase and Clone Mapper, respectively, with 23266 identified by both. This corresponded respectively to 1518, 3137 and 18664 individual RNAi clones. According to Wormbase annotations, half (49.8%) of the 3137 RNAi clones identified by Clone Mapper as potentially targeting a novel transcript (when using a cut-off score of ≥1) were previously predicted to target a single gene. As discussed below, the choice of cut-off is necessarily arbitrary, but our results, taken together with bioinformatic and experimental investigation of on- and off-target effects (Rual *et al.* 2007; Zhou *et al.* 2014), suggest that Clone Mapper can identify a substantial number of novel targets.

We also calculated the number of protein-coding genes targeted by the combined set of Vidal and Ahringer RNAi clones. Using the arbitrary cut-off score of ≥1, the entire set of clones is predicted to target a total of 19,120 of the 20,540 protein coding genes (93.1%; WS240). This figure only increases marginally, to 19,595 (95.4%), when the cut-off score is reduced to include all targets (Figure 5C).

**Figure 6:**
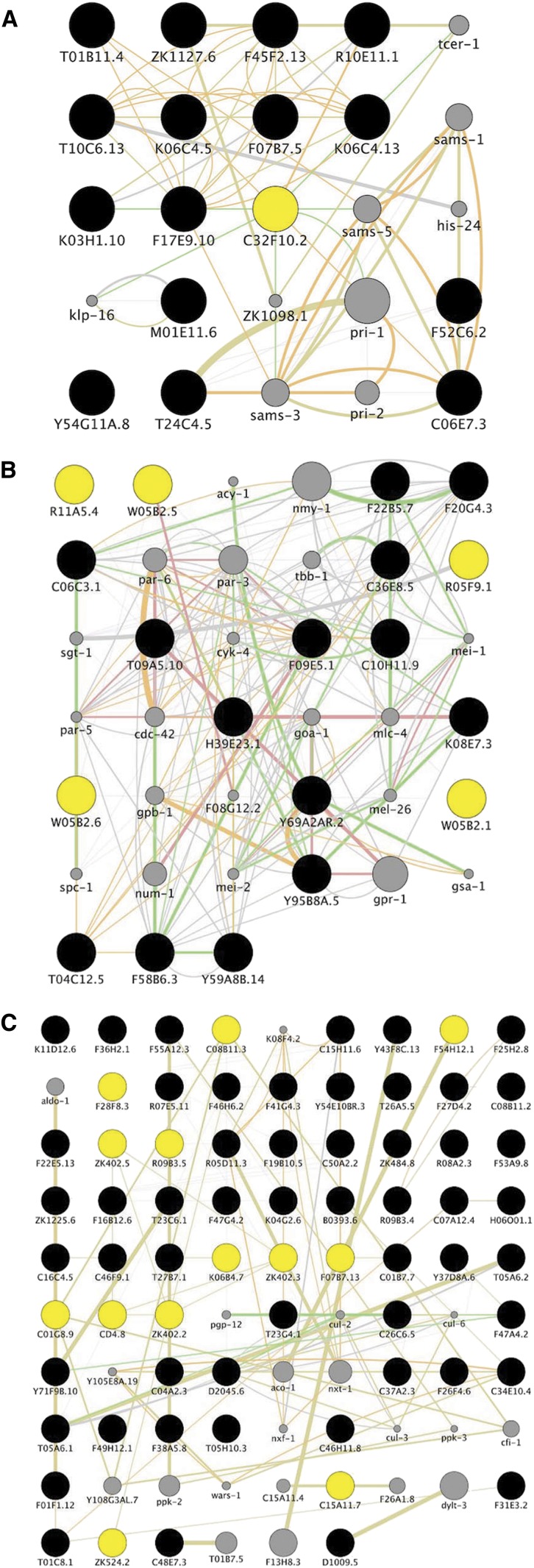
Network analysis of novel RNAi targets. (A) Ceron *et al.* undertook an RNAi screen to identify genes that interact with the *C. elegans* retinoblastoma gene *lin-35* (Ceron *et al.* 2007). The list of novel targets identified with Clone Mapper for the RNAi clones selected by Ceron *et al.* was used as input to GeneMania (black circles), together with *lin-35*/C32F10.2 (highlighted in yellow) as a seed gene. (B) Fievet *et al.* performed RNAi screens for *C. elegans* cell polarity mutants, to generate a polarity network (Fievet *et al.* 2013). A list of novel targets identified with Clone Mapper for the RNAi clones used by Fievet *et al.* was used as input to GeneMania (yellow circles), together with the genes corresponding to the 14 mutant strains used in the study (black circles). (C) Roy *et al.* performed a screen to find components of a regulatory network that promotes developmentally programmed cell-cycle quiescence (Roy *et al.* 2014). Novel targets identified with Clone Mapper for the RNAi clones used by Roy *et al.* (yellow), together with common targets (black) were used as input to GeneMania. The networks were trimmed to retain only direct neighbors; unconnected genes are not shown. Genes that are linked within GeneMania but do not appear on the list of RNAi clone targets are shown as gray circles; their size is proportional to the calculated probability score. Networks were displayed in Cytoscape; green edges represent experimentally-determined genetic interactions, pink edges represent experimentally-determined physical interactions for the corresponding proteins, orange and gray edges interactions predicted on the basis of co-expression or literature mining, respectively.

To evaluate the potential impact of these differences in prediction, we compared the list of putative targets in four published data sets with those obtained with Clone Mapper. In the first screen, where just 29 clones were selected (Pukkila-Worley *et al.* 2014), Clone Mapper predicted the same targets as published; no novel targets with high scores were identified. In the second specific case (Ceron *et al.* 2007), 14 of 244 targets were not predicted by Clone Mapper since the insert sequences of the corresponding clones cannot be predicted. On the other hand, Clone Mapper identified 23 new targets with of score >1, 9 of which had a score >50 (Supporting Information, Table S1). Similar results were obtained for the two other studies (Fievet *et al.* 2013; Roy *et al.* 2014) (Table 1, Table S2, and Table S3). In all cases, the novel targets identified with Clone Mapper formed part of a closely linked network (Figure 6). The interconnectivity of the novel RNAi targets suggests that they may be functionally important for the biological process under study. Such a hypothesis requires direct experimental validation, but the results demonstrate the potential utility of Clone Mapper in gene discovery.

**Table 1:** Identification of novel target genes with Clone Mapper

	(Pukkila-Worley *et al.* 2014)	(Roy *et al.* 2014)	(Fievet *et al.* 2013)	(Ceron *et al.* 2007)
Original number of target genes	29	102	436	245
False positive (when score >0)	0	18	11	66
False positive (when score >1)	0	19	33	66
New targets with score >0	38	25	400	84
New targets with score >1	0	24	9	73

### A comparison of Clone Mapper with available resources

Most published reports of RNAi experiments in *C. elegans* have relied on Wormbase for target identification. As explained previously, Wormbase has several limitations (Table 2). It does not include predictions for secondary targets for Vidal RNAi clones, and bases target identification on genomic DNA sequence, which is generally inappropriate for open reading frame−derived clones. This limitation has already been addressed in part by the web-based tool UP-TORR (Hu *et al.* 2013) that uses primer sequences to generate *in silico* a potential clone insert and then identify targets for that insert. UP-TORR, however, does not allow easy bulk clone-target mapping, or the use of the names of the Vidal library clones, for example. Furthermore, the current lower limit for stretches of sequence identity when searching for off-target genes with UP-TORR is 15 bp. This can expand the list of potential hits to an unmanageable size, especially since no score is ascribed to each clone-target pair. Clone Mapper addresses these different issues, and as a species-specific tool has been designed to be as simple and intuitive to use as possible.

**Table 2:** Comparison of tools for RNAi experiments

	Clone Mapper	Wormbase	UP-TORR
Clone verification	Yes	No	Yes
Tool for search	Mpscan	BLAT	Blast
Insert type	Genomic and cDNA	All genomic	Genomic and cDNA
All predicted clone inserts correspond to current Wormbase gene models	Yes	N/A	No
Flexible for match of primers to gene/transcript	Yes (perfect 10 bp match at 5′ or 3′ end sufficient).	N/A (uses pseudo-primers)	No
Secondary targets	Yes	Only sjj clones	Yes
Target score	Yes	No	No
Over-prediction	No	Yes	Yes
Under-prediction	No	Yes	Yes
Batch sequence retrieval	Yes	No	No
Optimal clone search	Yes	No	No

RNAi, RNA interference.

## Discussion

With Clone Mapper, we have attempted to satisfy several unmet needs for *C. elegans* researchers using RNAi. In addition to the central function of identifying potential targets for RNAi clones, it offers tools for clone verification and for the retrieval of RNAi clone and transcript sequences. Clone Mapper complements the tools already available in Wormbase and the web-based tool UP-TORR (Hu *et al.* 2013). It can be used in conjunction with Wormbase Converter (Engelmann *et al.* 2011) (also available via http://www.ciml.univ-mrs.fr/EWBANK_jonathan/software.html) to reanalyze published RNAi datasets. As with any resource, there are certain intrinsic and extrinsic limitations. A total of 1490 Vidal clones that are present in the physical library were purportedly amplified using primers that are not compatible with current gene models. In the example shown in Figure 1C, the mv_B0432.8 primers were used successfully to amplify a cDNA. Sequencing of this PCR product supports the existence of a transcript that spans B0432.8 and B0432.13. For a subset of Vidal library clones, it might thus be possible to reconstruct their insert sequences on the basis of OST data, but the OST coverage is incomplete (see for example, mv_B0432.9 in Figure 1C), and each case would require manual inspection. In common with UP-TORR, we therefore did not attempt to resolve these inconsistencies, nor did we try to evaluate systematically whether the current gene models in question are incorrect.

The ORFeome clones that were used to construct the Vidal RNAi library were generated by amplification of cDNA. Thus, for genes with more than one mRNA isoform, the corresponding clone may contain variants with inserts differing in one or more exon. As a consequence, even when sequence data are available for a given Vidal RNAi clone, one cannot exclude the possibility that multiple different inserts might be present since clones were not always completely sequenced (generally ca. 500 bp from 5′ and 3′ primers) and the prevalence of one splice variant may mask the presence of others (J. Reboul, personal communication).

Although we did not find any inconsistency between the publicly available sequence data and sequence data generated from our in-house library (n > 70; O. Zugasti, unpublished results), to be prudent, when constructing the *in silico* clone insert library, we assumed that each Vidal RNAi clone did contain inserts corresponding to every possible transcript. If in reality not all isoforms are represented in an RNAi clone, then there will be the potential for some over-prediction of off-target genes. When the clone insert sequence is known, it can be used as the input to Clone Mapper, thus avoiding this problem.

Within Clone Mapper, the length used to search for possible matches between clone insert and target transcript can be defined by the user, with a minimum of 6 bp, so that it can be used to identify potential seed regions for miRNAs (Grosswendt *et al.* 2014) in complete *C. elegans* transcripts. It can equally be increased to ensure specificity. The minimal length of sequence identity required to obtain efficient knock-down of green fluorescent protein expression in *C. elegans* has been experimentally determined to be ≥23 bp (Parrish *et al.* 2000). It has also been reported that to observe an efficient RNAi effect, the length may vary from 30 to 50 nucleotides (Rual *et al.* 2007). We set the default oligomer length at 21 bp since this is the size of a substantial proportion of siRNAs in *C. elegans* (Gent *et al.* 2010). Increasing oligomer length will obviously reduce the number of potential targets, whereas decreasing it will broaden the set of potential targets. The different targets are assigned scores that help in the evaluation of whether a transcript is likely to be a high-confidence target. It also permits users to evaluate the consequences of setting different values for these parameters. To be inclusive but selective, one could decrease oligomer length and then set a high cut-off score. There is an element of arbitrariness in choosing oligomer length and cut-off scores, but this reflects a biological reality. The efficiency with which a given transcript is knocked down depends not only on its sequence, but also on the level at which it is expressed, the tissue that it is expressed in, and on the expression of any other transcripts that share sequence with it. Indeed, siRNAs generated from a diced primary target (secondary siRNAs) can knock-down mRNAs that are not a direct target of siRNA derived from an RNAi clone (Zhou *et al.* 2014). We did not implement this level of target identification as part of the tool, but users can search for these indirect hits by inputting the sequence of any target transcript into the *de novo* target prediction utility that is available within Clone Mapper.

The modular architecture of Clone Mapper also allows users to choose the best reagent for specifically knocking down a given gene. The identity of clones predicted to target a given gene, or set of genes, can be retrieved. Then one can check the number of off-target genes predicted for each clone, to identify the most specific clone.

Finally, an *in silico* reanalysis of selected published RNAi datasets identified new target genes. The demonstration of the functional relevance of these targets is beyond the scope of this study, but these results illustrate Clone Mapper’s potential for gene discovery.

## Supplementary Material

Supporting Information
