# Supplementary material for: Clone Mapper: An Online Suite of Tools for RNAi Experiments in Caenorhabditis elegans
Source: G3 (Bethesda). 2014 Sep 2;4(11):2137–45. doi: 10.1534/g3.114.013052 (PMC4232539; doi:10.1534/g3.114.013052)
Supplement: Supporting Information [file supp_4_11_2137__index.html]

Clone Mapper: An Online Suite of Tools for RNAi Experiments in Caenorhabditis elegans — Supporting Information 

# Clone Mapper: An Online Suite of Tools for RNAi Experiments in *Caenorhabditis elegans*

## Supporting Information for Thakur *et al.*, 2014

**Files in this Data Supplement:**

- Table S1 - Raw data associated with Fig. 6a (.xls, 614 KB)
- Table S2 - Raw data associated with Fig. 6b (.xls, 9 MB)
- Table S3 - Raw data associated with Fig. 6c (.xls, 4 MB)
